# Supplementary material for: Gene therapy conversion of striatal astrocytes into GABAergic neurons in mouse models of Huntington’s disease
Source: Nat Commun. 2020 Feb 27;11:1105. doi: 10.1038/s41467-020-14855-3 (PMC7046613; doi:10.1038/s41467-020-14855-3)
Supplement: Supplementary file 1 — Supplementary Information [file 41467_2020_14855_MOESM1_ESM.pdf]

Supplementary information

Gene therapy conversion of striatal astrocytes into GABAergic neurons in mouse models of Huntington's disease

Wu et al.

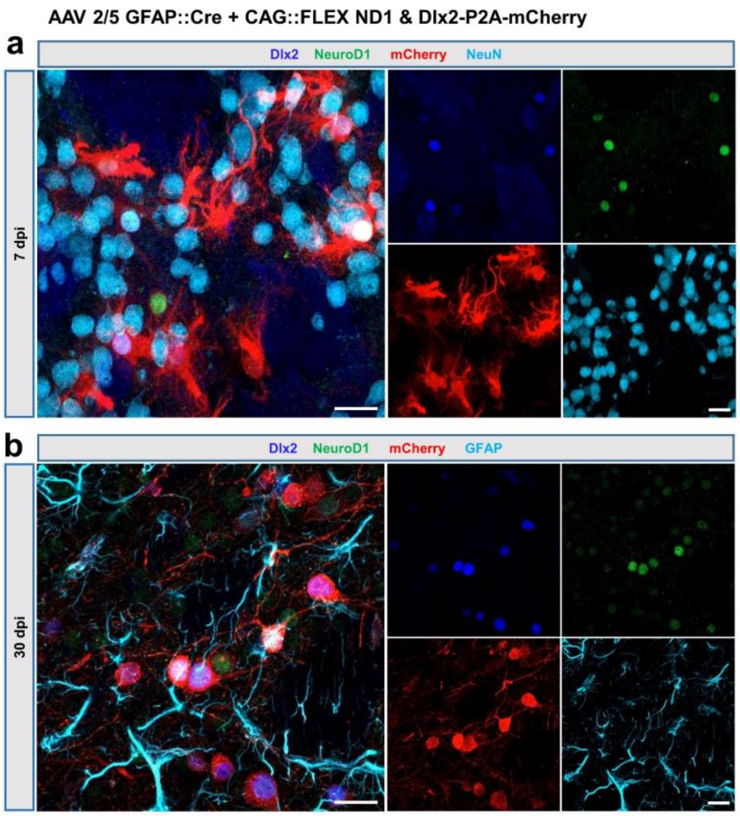

Supplementary Figure 1. Ectopic expression of NeuroD1 and Dlx2 in AAV-infected cells.

(**a**) Co-staining of Dlx2 (dark blue), NeuroD1 (green), mCherry (red), and NeuN (cyan) at 7 days post AAV2/5 injection (7 dpi). No NeuroD1 or Dlx2 were detected in NeuN positive cells at 7 dpi. (**b**) Co-staining of Dlx2 (dark blue), NeuroD1 (green), mCherry (red), and GFAP (cyan) at 30 days post AAV2/5 injection (30 dpi). NeuroD1 and Dlx2 were colocalized with mCherry, but not with GFAP. Scale bar: 20 µm. Quantification was shown in **Figure 2c**.

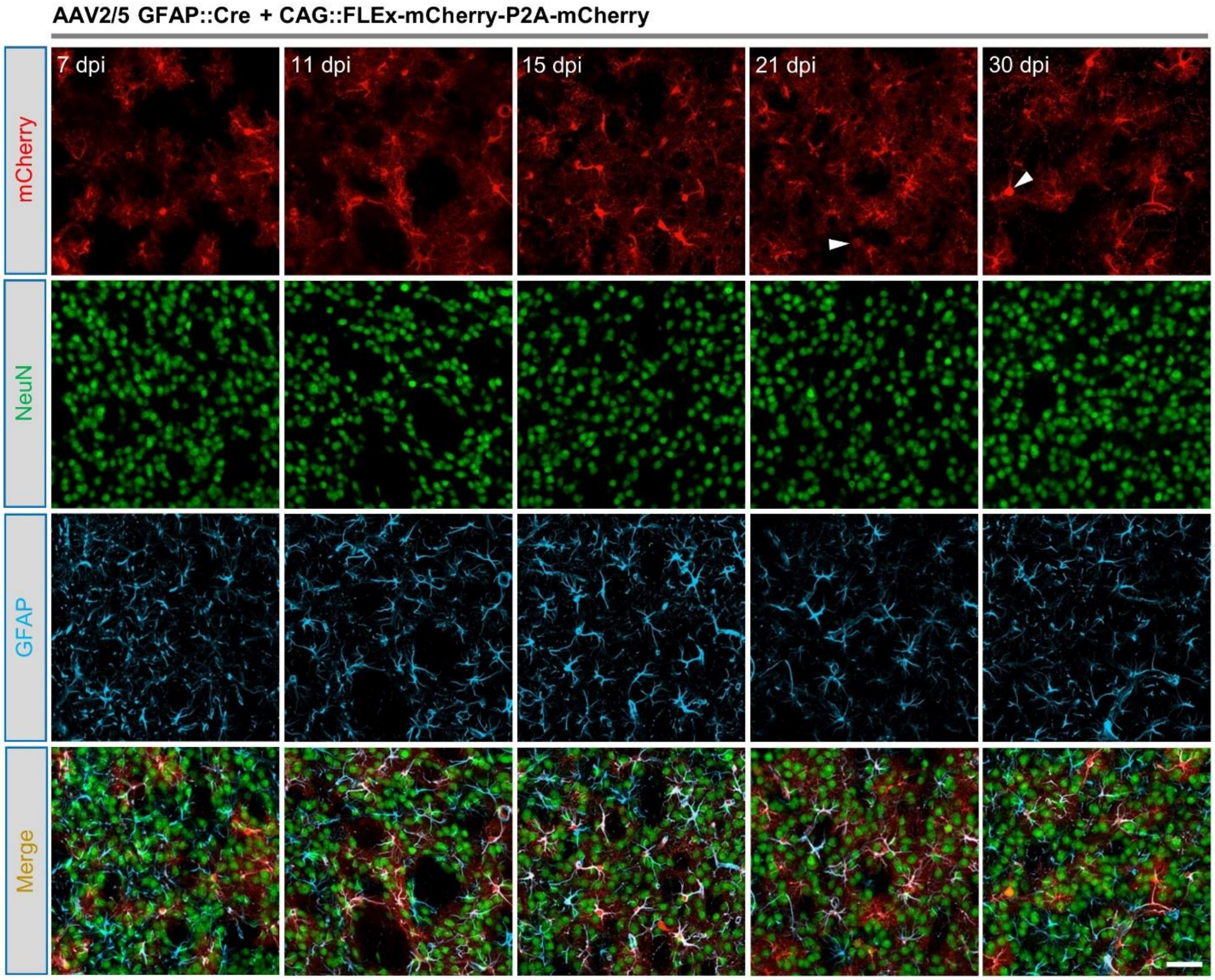

Supplementary Figure 2. Time course of mCherry control virus infection in the striatum of WT mice.

WT mice were injected with AAV2/5 GFAP::Cre + AAV2/5 CAG::mCherry-P2A-mCherry, and sacrificed at different time points (7, 11, 15, 21, and 30 dpi) for immunohistochemistry analyses. Most of the mCherry$^+$ cells co-stained with GFAP but not NeuN, with only a few exception at 21 and 30 dpi (arrowhead). Scale bar: 50 µm. Quantification was shown in **Figure 2f**.

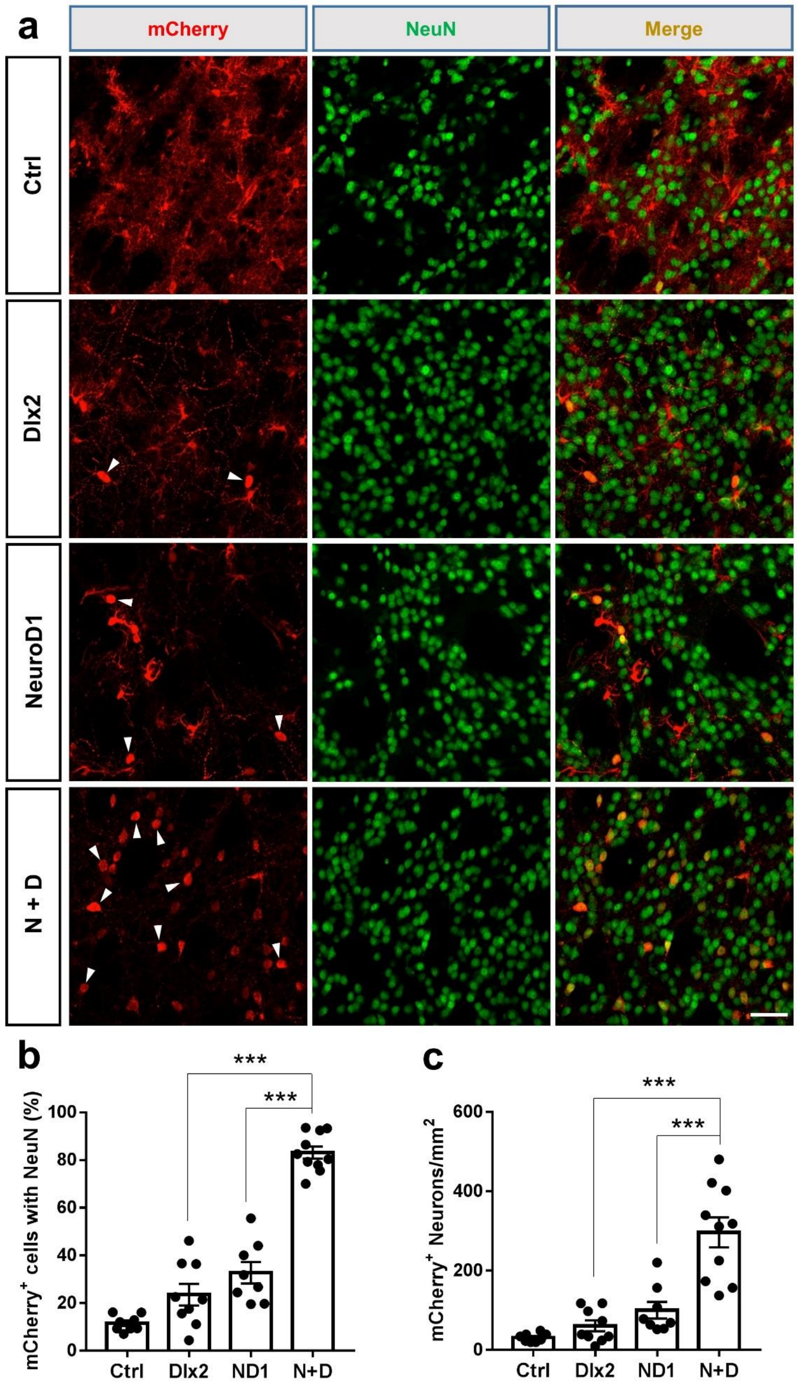

Supplementary Figure 3. Synergistic effect of NeuroD1 and Dlx2 in increasing the conversion efficiency in the striatum.

(**a**) WT mice were injected with different AAV2/5 and sacrificed at 30 dpi for immunostaining analysis to compare the conversion efficiency among different groups. Scale bar: 50 μm. (**b, c**) Quantified data showing that the NeuroD1 plus Dlx2 group has the highest conversion efficiency (**b**) and generates the greatest number of neurons (**c**). Data are shown as mean ± SEM.

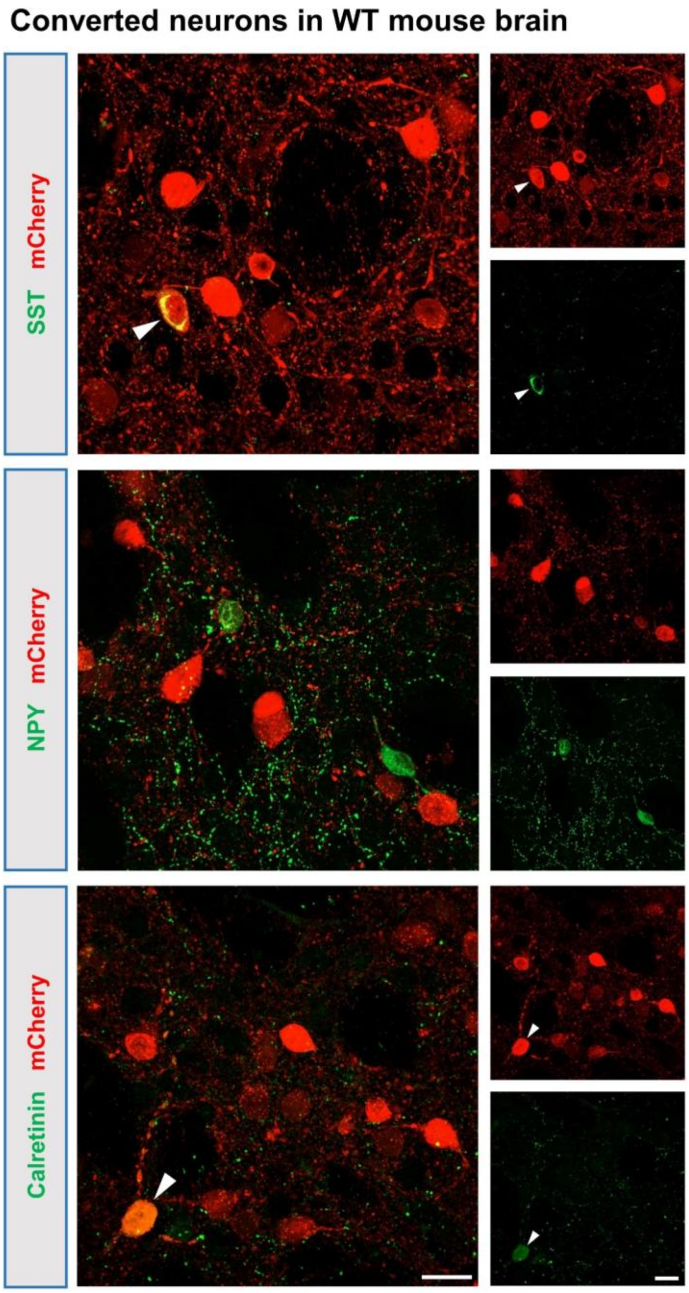

Supplementary Figure 4. Neuronal subtype characterization among the striatal astrocyte-converted neurons in the WT mouse striatum.

The mouse brain sections were co-stained with different GABAergic subtype markers at 30 dpi. Few converted neurons were positive for somatostatin (SST, green), neuropeptide Y (NPY, green), or calretinin (green). Scale bar: 20 µm. Quantified data were shown in **Figure 2h**.

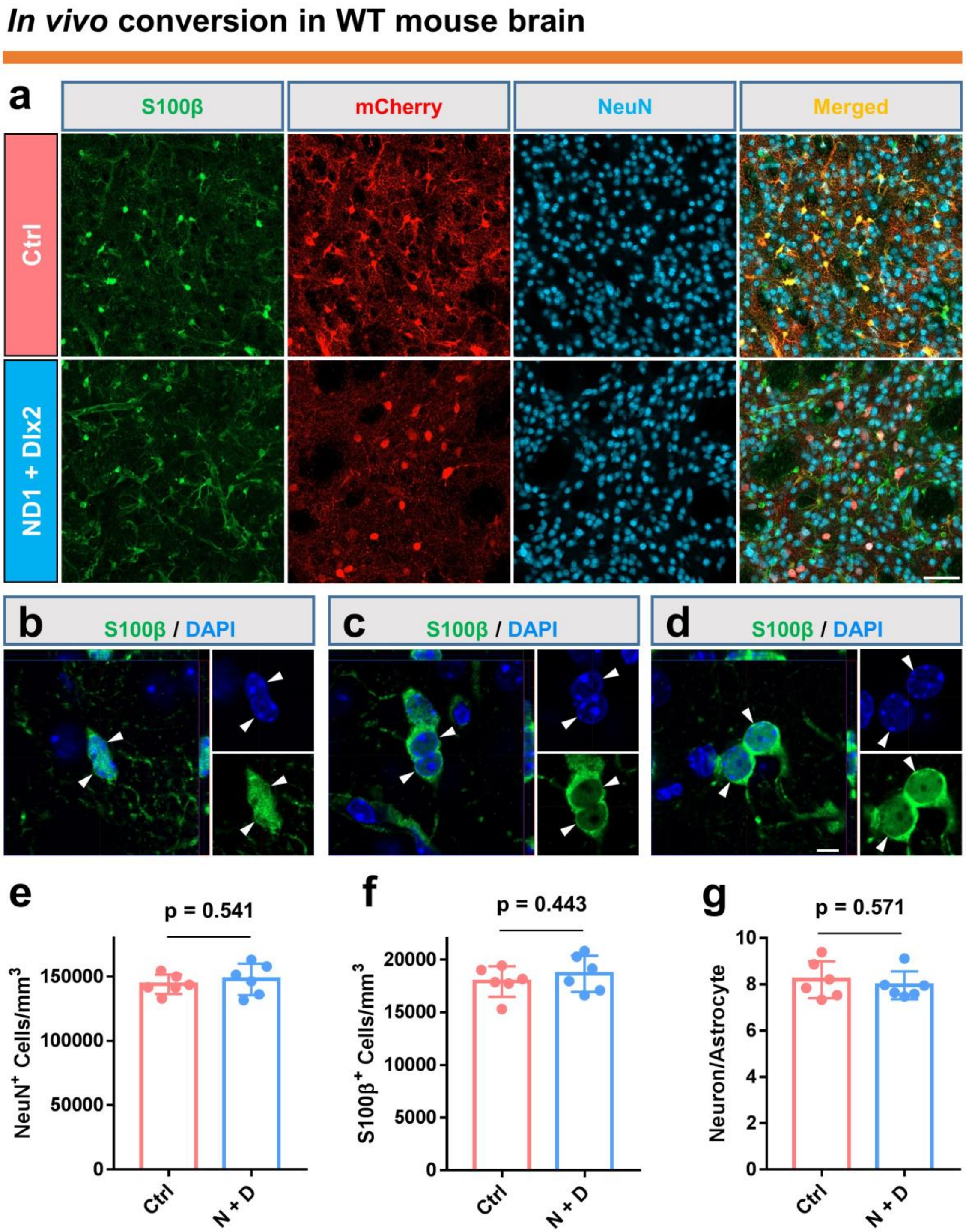

Supplementary Figure 5. Striatal neuron and astrocyte density in WT mouse brain after conversion.

(**a**) Confocal images showing the astrocytic marker S100β (green) and neuronal marker NeuN (cyan) at 30 days post AAV injection. Scale bar: 20 µm. (**b-d**) High magnification confocal

images showing dividing astrocytes at different stages found in NeuroD1 + Dlx2 treated mouse brains, indicating astrocytic proliferation after conversion. (**e-g**) Summary graphs showing neuronal density (**e**), astrocytic density (**f**), and the ratio of neuron/astrocyte (**g**) in control condition (red bar) or after cell conversion (blue bar), with no significant difference. Data are shown as mean ± SD.

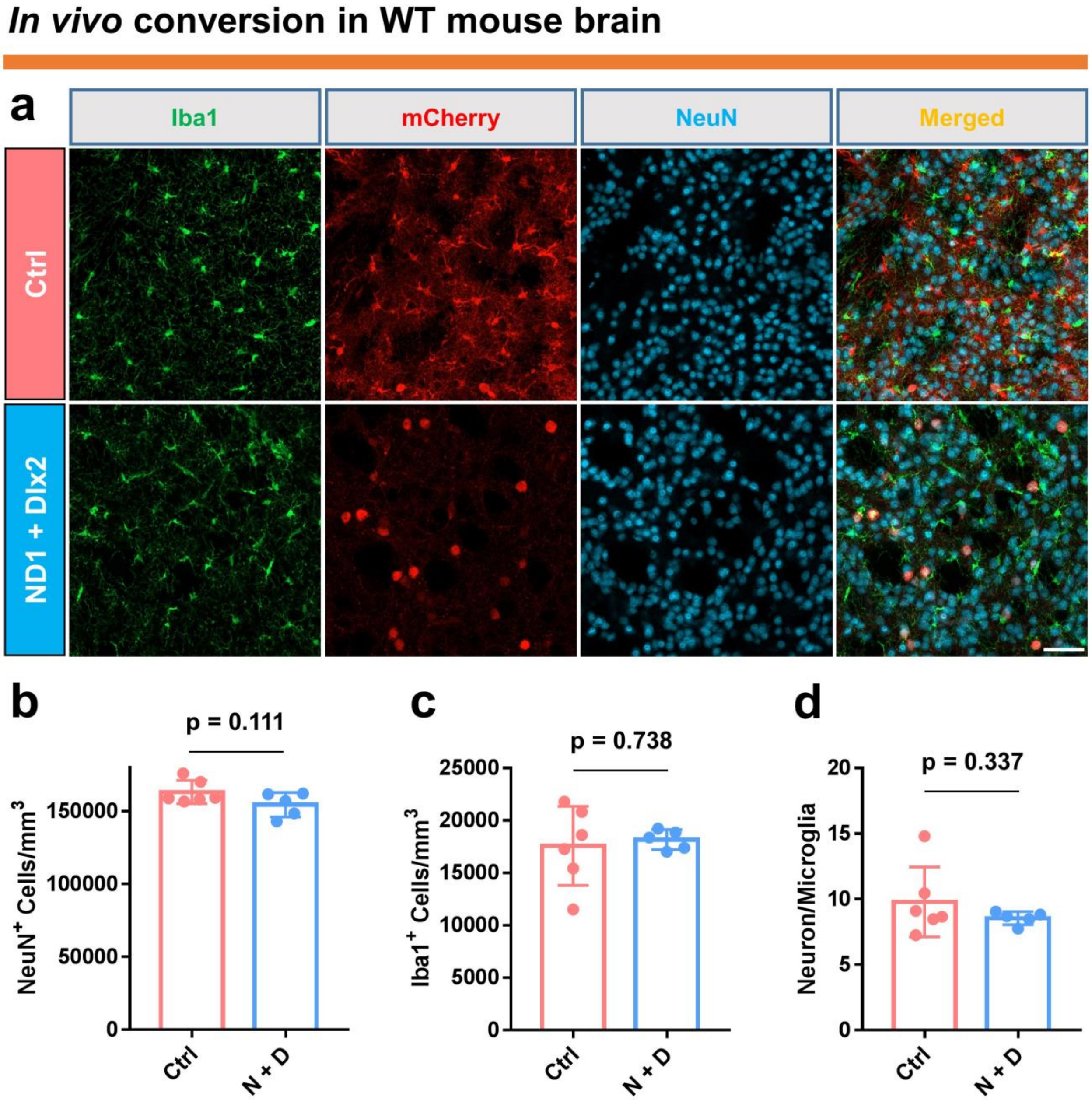

Supplementary Figure 6. Striatal neuron and microglia density in WT mouse brain after cell conversion.

(**a**) Confocal images showing the microglial marker Iba1 (green) and neuronal marker NeuN (cyan) at 30 days post AAV injection. Scale bar: 20 µm. (**b-d**) Summary graphs showing neuronal density (**b**), microglial density (**c**) and the ratio of neuron/microglia (**d**) not changed after cell conversion. Data are shown as mean ± SD.

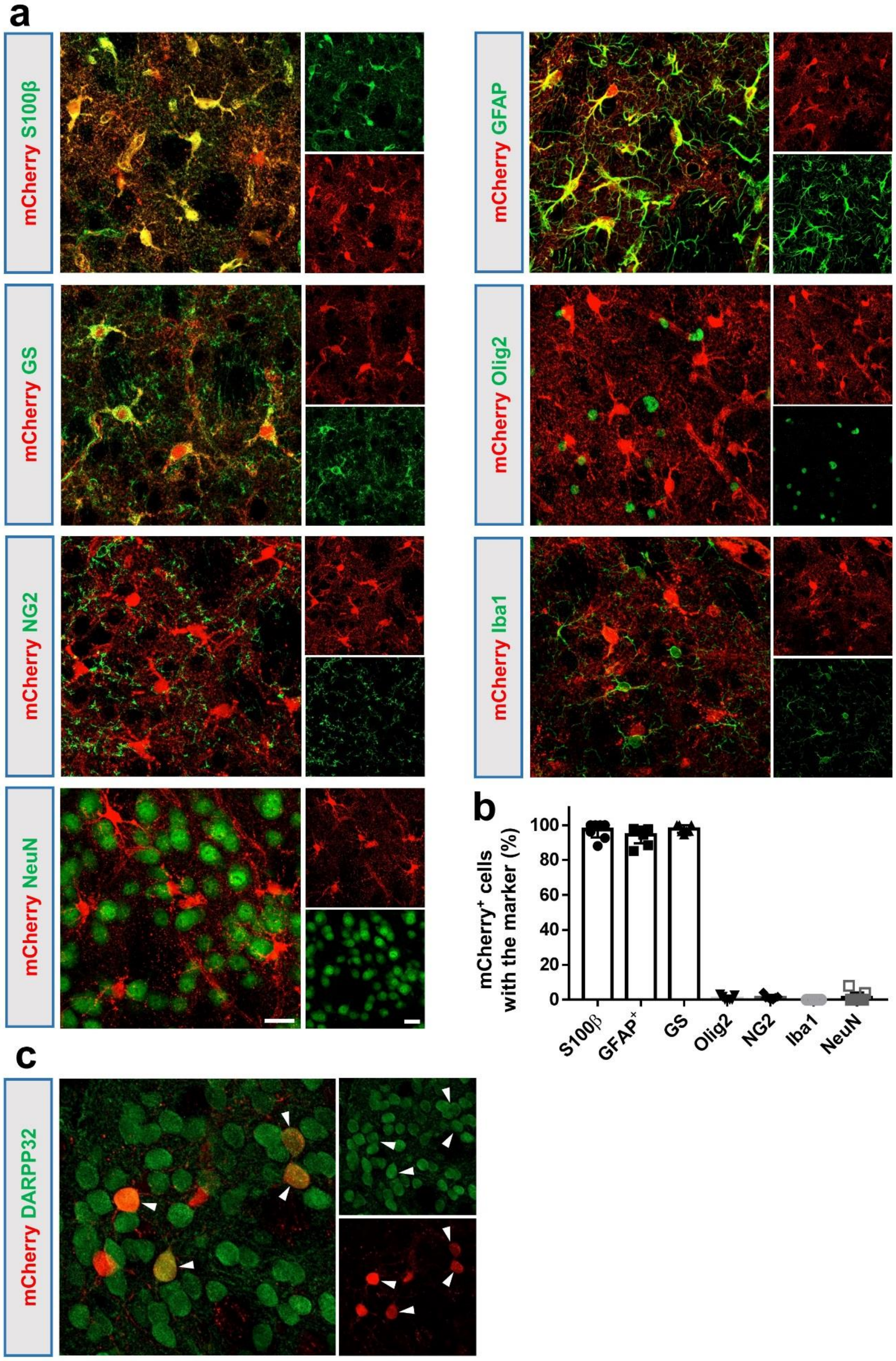

Supplementary Figure 7. Targeting striatal astrocytes for neuronal conversion in the GFAP::Cre77.6 transgenic mouse line.

(**a**) Confocal images showing the control AAV mCherry-infected cells in the striatum co-staining with different glial markers and neuronal marker at 58 dpi. Most of the mCherry$^+$ cells were co-localized with astrocytic markers including S100β, GFAP, and glutamine synthetase (GS). Very

few $mCherry^+$ cells co-stained with Olig2, NG2, Iba1, or NeuN. Scale bar: 20 µm. (**b**) Quantified data of (**a**) showing the percentage of the $mCherry^+$ cells that co-stained with different markers. Over 95% of $mCherry^+$ cells were positive for astrocyte markers in the striatum of GFAP::Cre 77.6 mouse line. (**c**) In NeuroD1 + Dlx2-treated striatum of the GFAP::Cre 77.6 mouse line, the majority of the astrocyte-converted neurons were immunopositive for DARPP32 (58 dpi). Scale bar: 20 µm. Data are shown as mean ± SEM.

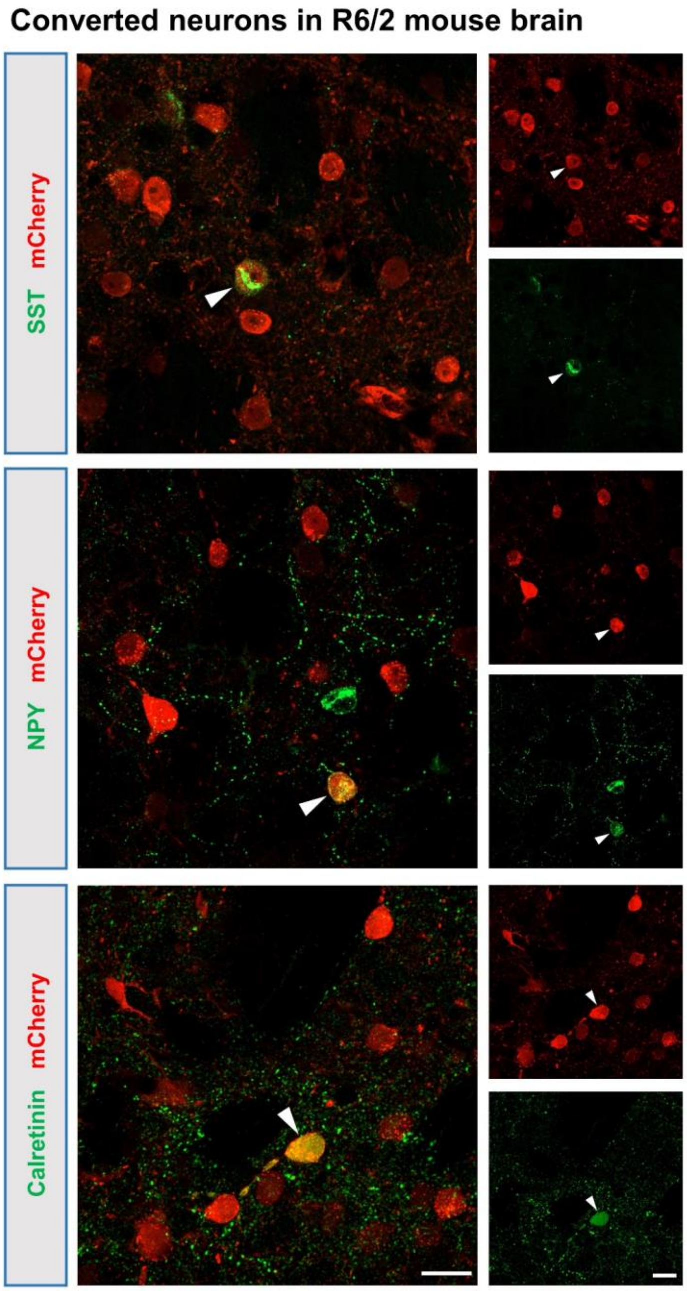

Supplementary Figure 8. Subtype characterization of converted neurons in the R6/2 mouse striatum.

Among the striatal astrocyte-converted neurons following NeuroD1 + Dlx2 treatment in the R6/2 mouse striatum, only a few of the converted neurons were immunopositive for somatostatin (SST), neuropeptide Y (NPY), or calretinin. Scale bar: 20 μm. Quantification was shown in **Figure 4f**.

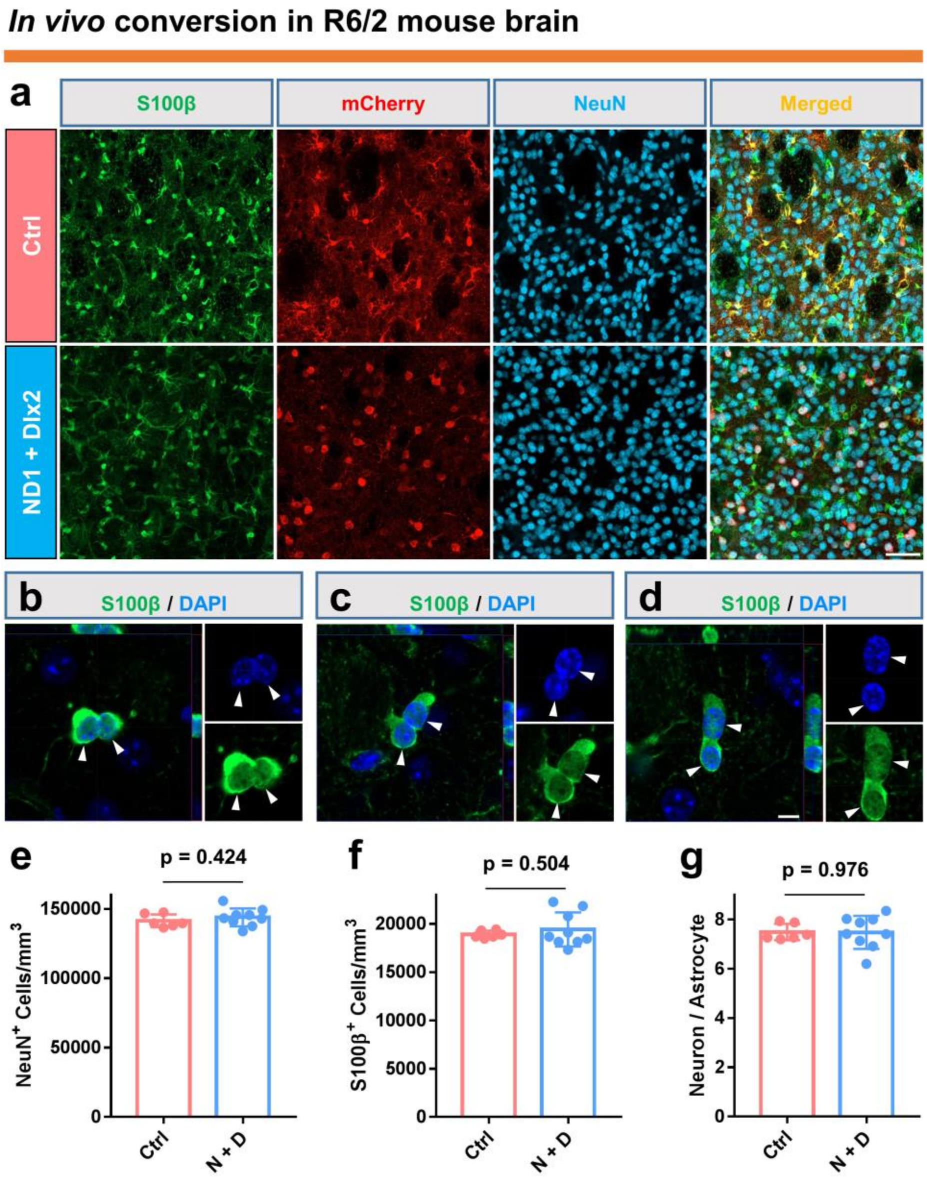

Supplementary Figure 9. Striatal neuron and astrocyte density in R6/2 mouse brains after cell conversion.

(**a**) Typical confocal images of astrocytes (green), AAV-infected cells (red) and neurons (cyan) in R6/2 mouse striatum at 30 days after viral injection. Scale bar: 20 µm. (**b-d**) High magnification confocal images showing different stages of dividing astrocytes in R6/2 mouse striatum after NeuroD1 + Dlx2 treatment, indicating astrocytic proliferation after conversion. (**e-g**) Summary graphs illustrating neuronal density (**e**), astrocytic density (**f**), and the ratio of neuron/astrocyte (**g**) in the R6/2 mouse striatum without (red bar) or with cell conversion (blue bar). Data are shown as mean ± SD.

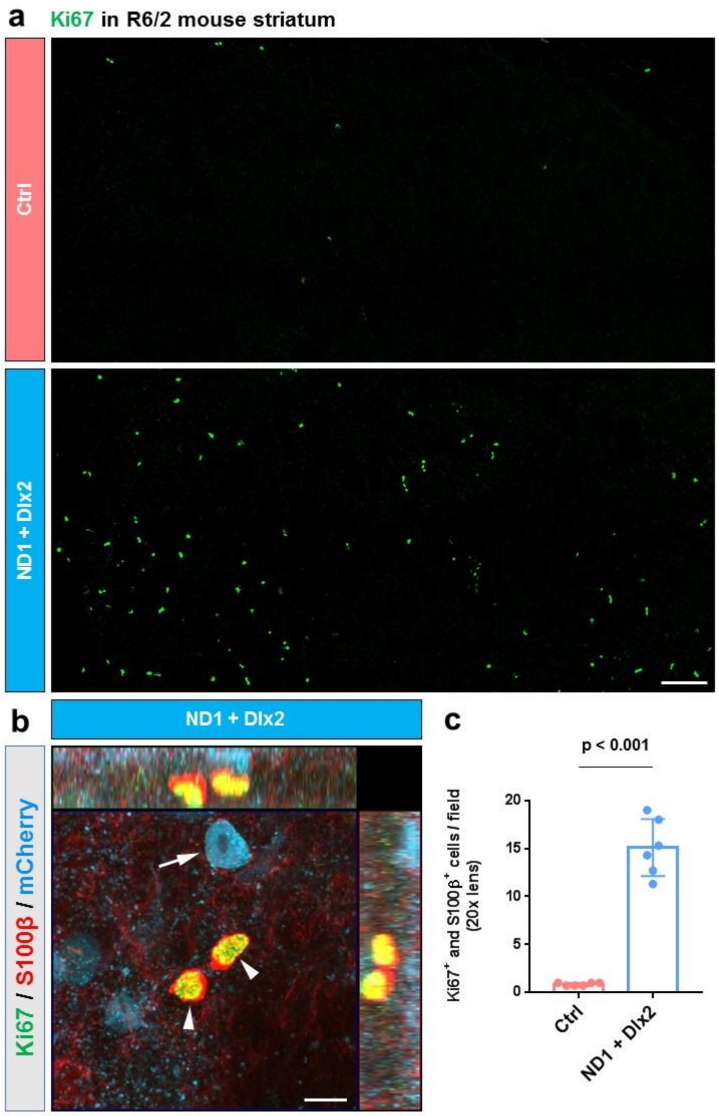

Supplementary Figure 10. Cell conversion triggers proliferation of striatal astrocytes in R6/2 mouse brains.

(**a**) Tiled low magnification confocal images of Ki67 immunostaining (green) showing many proliferating cells detected in the NeuroD1 + Dlx2-treated R6/2 mouse striatum, but very few in the striatum of control AAV-treated R6/2 mice. Scale bar: 100 μm. (**b**) High magnification confocal images showing proliferating astrocytes (arrowheads) in R6/2 mouse striatum after NeuroD1 + Dlx2 treatment. The arrow indicates a converted neuron (cyan, pseudo-color). Scale bar: 10 μm. (**c**) Summary graph showing the number of proliferating astrocytes dramatically increased in NeuroD1 + Dlx2-treated R6/2 striatum (30 dpi), suggesting that in vivo astrocyte-to-neuron conversion can significantly stimulate the proliferation of astrocytes to replenish themselves after astrocyte conversion. Data are shown as mean ± SD.

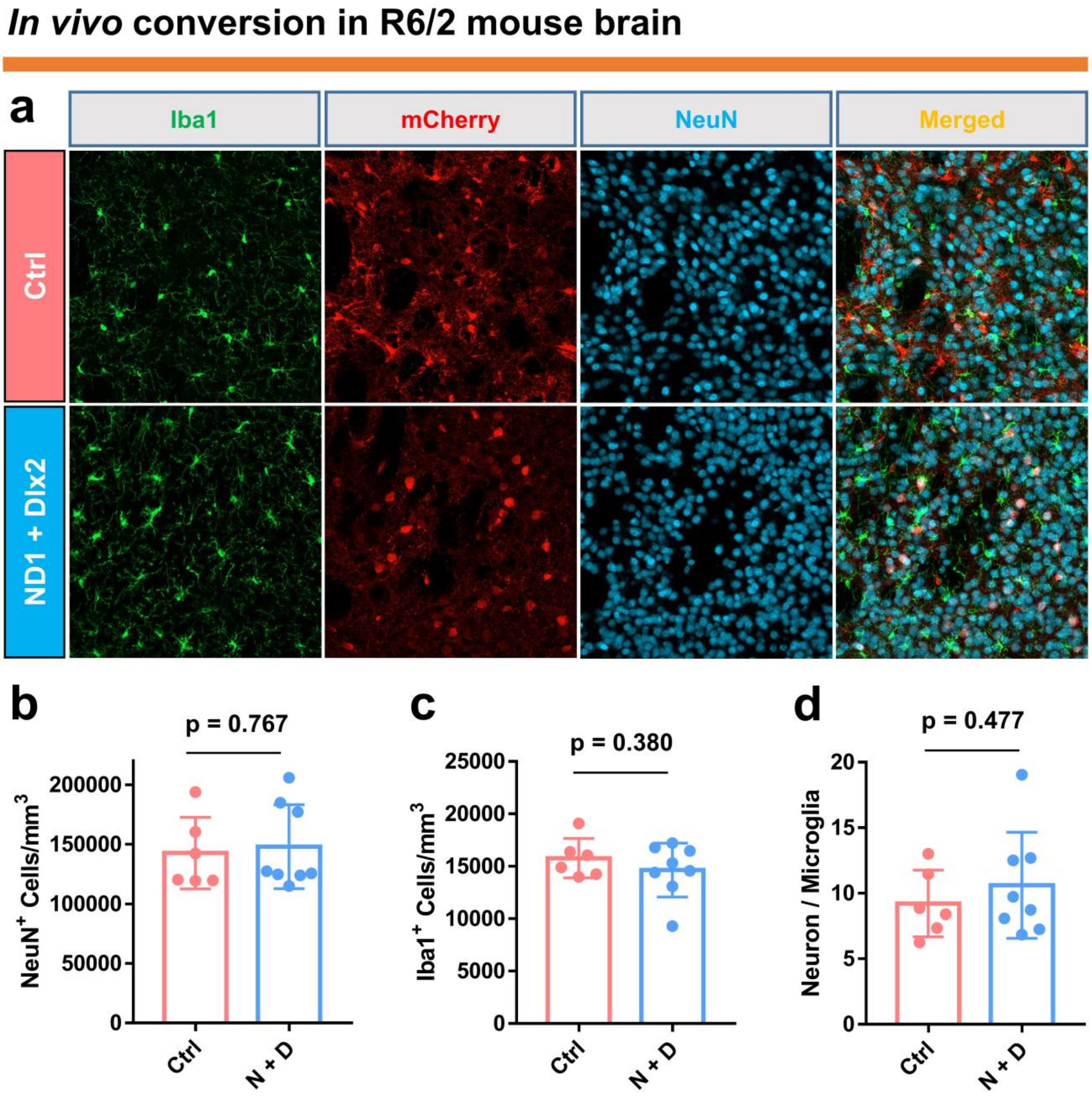

Supplementary Figure 11. Striatal neuron and microglia density in R6/2 mouse brains after cell conversion.

(**a**) Confocal images showing the microglial marker Iba1 (green) and neuronal marker NeuN (cyan) at 30 days post AAV injection. Scale bar: 20 µm. (b-**d**) Summary graphs showing neuronal density (**b**), microglial density (**c**), and the ratio of neuron/microglia (**d**) in control (red bar) and NeuroD1+Dlx2 group (blue bar). Data are shown as mean ± SD.

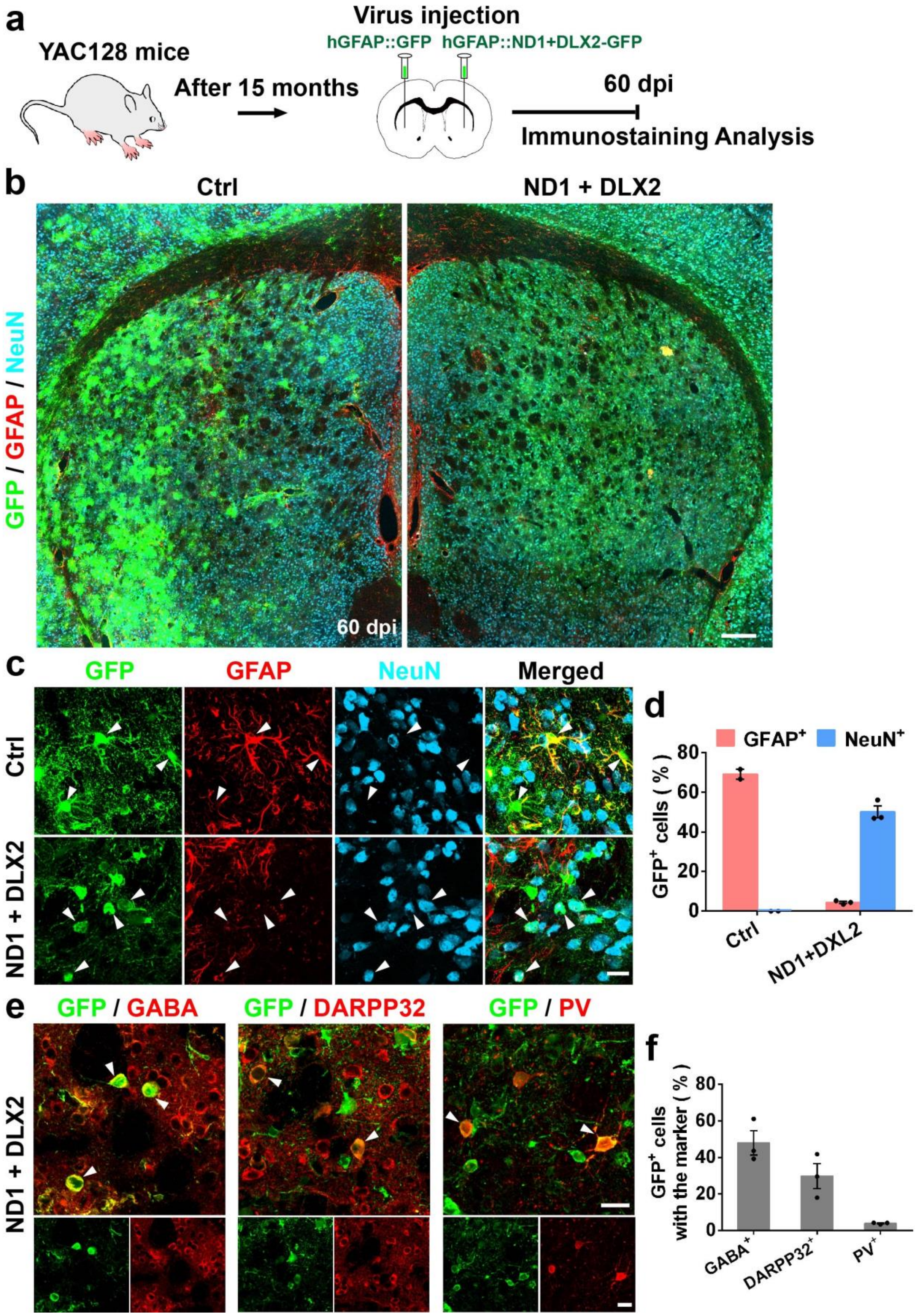
a
YAC128 mice
After 15 months
Virus injection
hGFAP::GFP
hGFAP::ND1+DLX2-GFP
60 dpi
Immunostaining Analysis
b
Ctrl
ND1 + DLX2
GFP / GFAP / NeuN
60 dpi
c
GFP
GFAP
NeuN
Merged
Ctrl
ND1 + DLX2
d
GFAP⁺
NeuN⁺
GFP⁺ cells (%)
80
60
40
20
0
Ctrl
ND1+DXL2
e
GFP / GABA
GFP / DARPP32
GFP / PV
ND1 + DLX2
f
GFP⁺ cells with the marker (%)
80
60
40
20
0
GABA⁺
DARPP32⁺
PV⁺

Supplementary Figure 12. In vivo conversion of striatal astrocytes into GABAergic neurons in the YAC128 mouse brains.

(**a**) Experimental design of testing cell conversion in 15-month old YAC128 transgenic mice. Expression of NeuroD1 and Dlx2 were directly driven by the hGFAP promotor. (**b**) Low-magnification coronal section view of the YAC128 mouse striatum injected with control GFP AAV (left panel) or NeuroD1 + Dlx2 AAV (right panel) at 60 dpi. Scale bar: 0.5 mm. (**c**) High-magnification images of GFP^+ infected cells co-stained with GFAP (red) and NeuN (cyan). Arrowheads indicate GFP^+ cells co-labeled with GFAP in the control group (top row), and co-labeled with NeuN in the NeuroD1 + Dlx2 treated group (bottom row). Scale bar: 20 μm. (**d**) Summary data showing that by 60 dpi, the majority of GFP^+ cells in the control group were remaining $GFAP^+$ astrocytes; but in the NeuroD1 + Dlx2 group, half of the GFP^+ cells were converted into $NeuN^+$ neurons. Data are shown as mean ± SEM. (**e**) Confocal images showing converted neurons in the YAC128 mouse striatum co-labeled with GABA (left), DARPP32 (middle), and parvalbumin (right). (**f**) Summary graph showing the percentage of converted neurons in the YAC128 mouse striatum shown in panel (**e**).

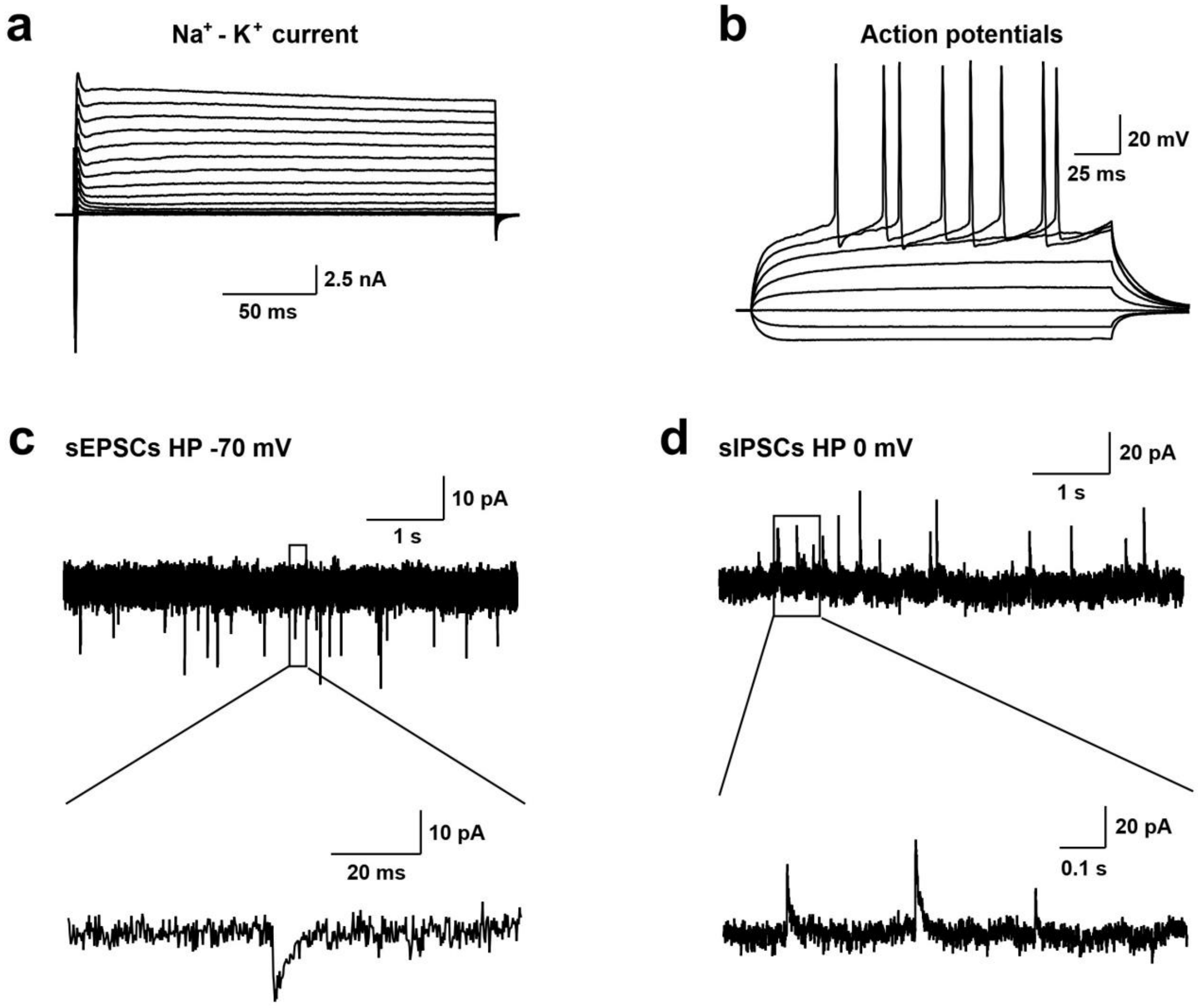

Supplementary Figure 13. Typical electrophysiological traces recorded from striatal neurons in the wild type mice.

(**a**) Representative traces showing Na^{+}-K^{+} currents recorded from striatal neurons in the wild type mouse. (**b**) Typical traces of action potentials recorded from WT striatal neurons. (**c-d**) Typical traces of spontaneous EPSCs (**c**) and spontaneous IPSCs (**d**) recorded from WT striatal neurons.

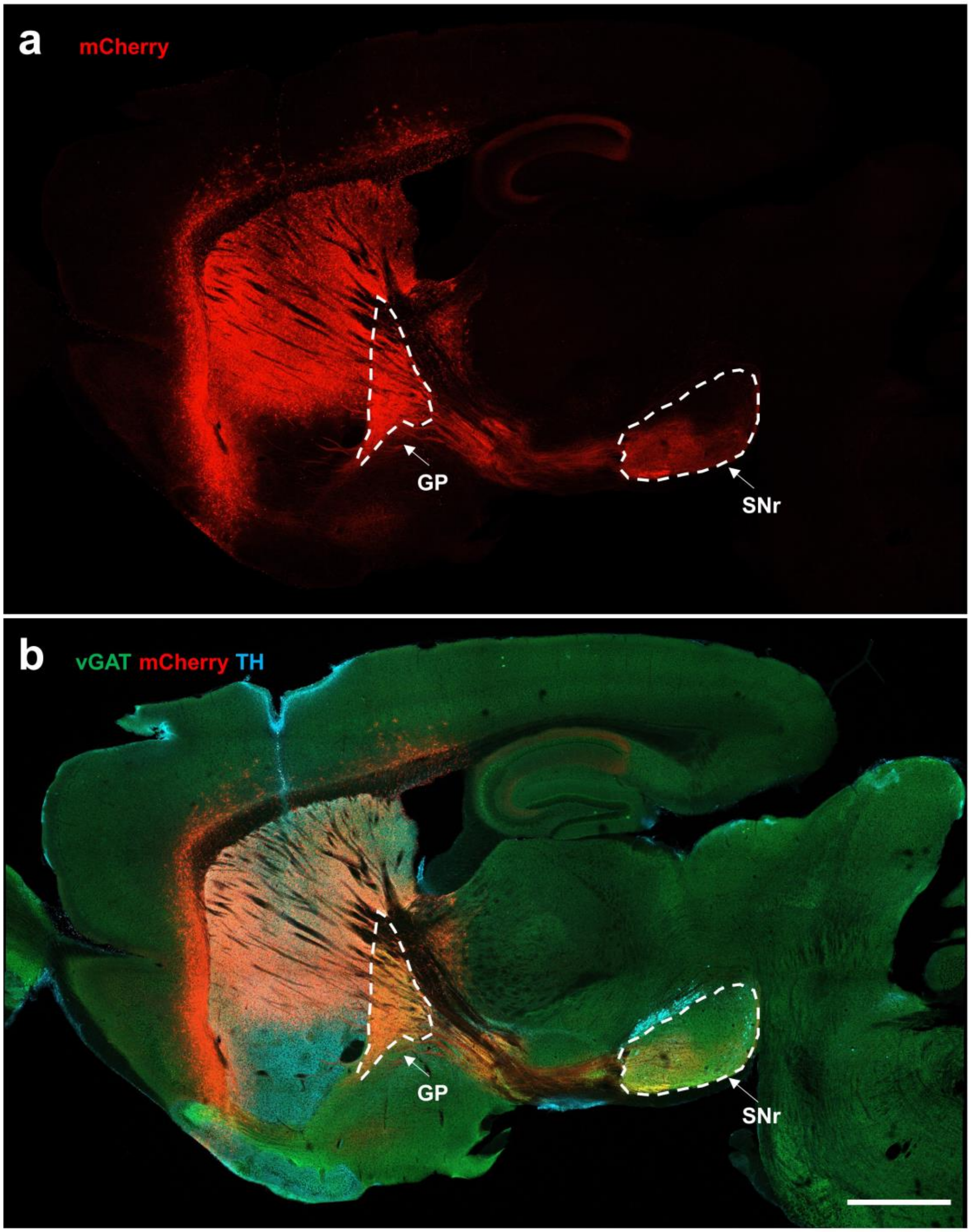

Supplementary Figure 14. Sagittal view of R6/2 mouse brain showing axonal projection from newly converted neurons post NeuroD1 + Dlx2 treatment.

(**a**) Tiled image showing sagittal view of R6/2 mouse brain at 38 days post viral injection of NeuroD1+Dlx2. mCherry^{+} converted neurons sent axonal projections to GP and SNr areas. (**b**) Merged images showing the mCherry signal relative to other brain regions. Scale bar: 1 mm. **This is enlarged view of Figure 6a.**

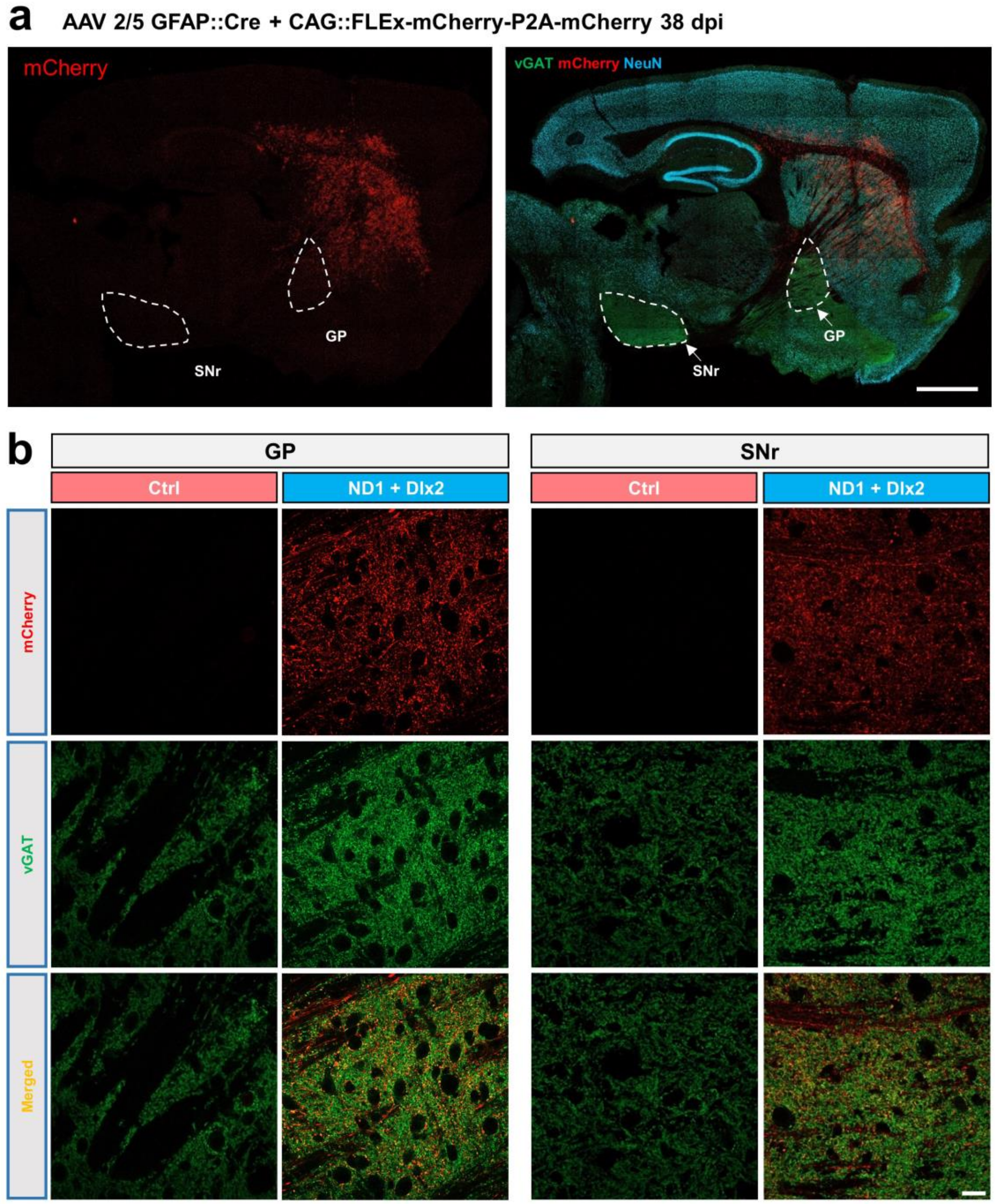

Supplementary Figure 15. Axonal projections of the striatal astrocyte-converted neurons in the R6/2 mouse brain.

(**a**) Tiled image showing sagittal view of an R6/2 mouse brain injected with control AAV mCherry (38 dpi). No mCherry^{+} signal detected in the GP or SNr. Scale bar: 1 mm. (**b**) High-magnification images showing a significant axonal projection from converted neurons (mCherry^{+} signal in ND1+Dlx2) to the target areas of GP and SNr following NeuroD1 plus Dlx2 treatment (38 dpi), but no mCherry^{+} signal detected in the GP and SNr after control virus treatment (38

dpi). Scale bar: 10 µm. The high-resolution images of the mCherry and vGAT puncta were shown in **Figure 6b** and quantified data were shown in **Figure 6c**.

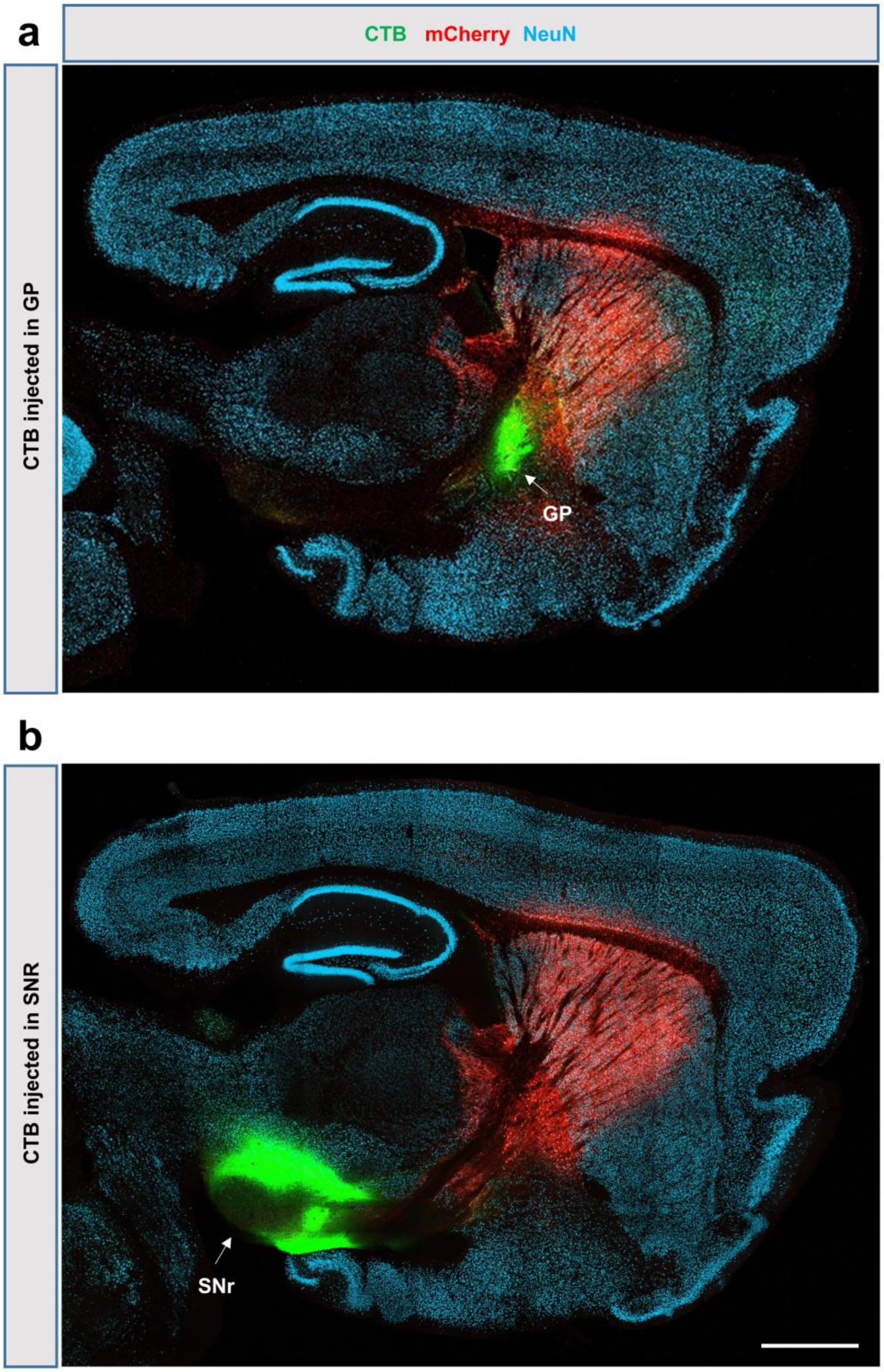

Supplementary Figure 16. Validating the sites of CTB injection.

(**a**) A sagittal view of CTB (green) injection in the GP. (**b**) A sagittal view of CTB (green) injection in the SNr. Mice were sacrificed at 7 days post CTB injection. Scale bar: 1 mm.

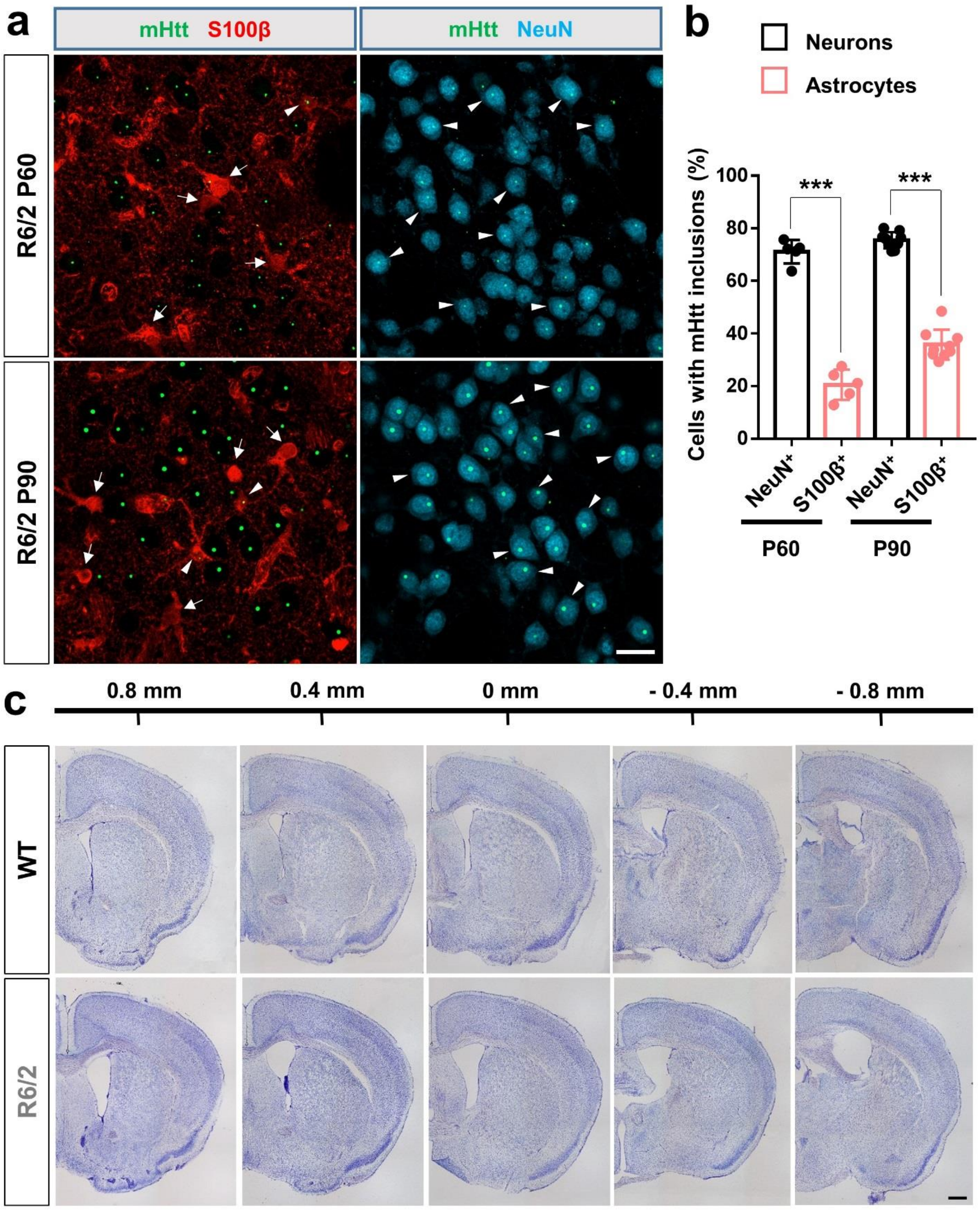

Supplementary Figure 17. mHtt inclusions and striatum atrophy in non-surgery R6/2 mice. (**a**) In non-surgery R6/2 mice, mHtt inclusions (green) were mostly found in striatal neurons (NeuN, cyan) and less in astrocytes (S100β, red) (age of P60 and P90). Arrows indicate cells without mHtt inclusions and Arrowheads indicate cells with mHtt inclusions. Scale bar: 20 µm. (**b**) Quantified data of (**a**). (**c**) Nissl staining of serial coronal sections of WT littermates and R6/2

mice without surgery (age of P90). Scale bar: 0.5 mm. The quantified data were shown **in Figure 7d**.
