# Supplementary material for: Gene therapy conversion of striatal astrocytes into GABAergic neurons in mouse models of Huntington’s disease
Source: Nat Commun. 2020 Feb 27;11:1105. doi: 10.1038/s41467-020-14855-3 (PMC7046613; doi:10.1038/s41467-020-14855-3)
Supplement: Supplementary file 3 — Description of Additional Supplementary Information [file 41467_2020_14855_MOESM3_ESM.pdf]

Description of Additional Supplementary Files

File Name: Supplementary Movie 1
Description: A movie of a WT littermate (age P92) walking in the catwalk setup tunnel.

File Name: Supplementary Movie 2
Description: A movie of a R6/2 mouse (age P92) walking in the catwalk setup.

File Name: Supplementary Movie 3
Description: A typical video of R6/2 mouse that were received mCherry control virus injection. Virus was injected at age of 2 month and the behavior test was performed at 34 days post AAV injection.

File Name: Supplementary Movie 4
Description: Catwalk behavior test movie of R6/2 mouse that were treated by NeuroD1 + Dlx2 AAV. Virus was injected at age of 2 month and the behavior test was performed at 34 days post AAV injection.

File Name: Supplementary Movie 5
Description: A clasping behavior test video of a R6/2 mouse was treated by control virus. AAV was injected at age of 2 month and the behavior test was performed at 30 days post AAV injection.

File Name: Supplementary Movie 6
Description: A clasping behavior test video of a R6/2 mouse was treated by NeuroD1 + Dlx2. AAV was injected at age of 2 month and the behavior test was performed at 30 days post AAV injection.
