# Supplementary material for: Gene therapy conversion of striatal astrocytes into GABAergic neurons in mouse models of Huntington’s disease
Source: Nat Commun. 2020 Feb 27;11:1105. doi: 10.1038/s41467-020-14855-3 (PMC7046613; doi:10.1038/s41467-020-14855-3)
Supplement: Supplementary file 10 — Reporting summary [file 41467_2020_14855_MOESM10_ESM.pdf]

nature research

Corresponding author(s): Gong Chen

Last updated by author(s): Oct 15, 2019

Reporting Summary

Nature Research wishes to improve the reproducibility of the work that we publish. This form provides structure for consistency and transparency in reporting. For further information on Nature Research policies, see Authors & Referees and the Editorial Policy Checklist .

Statistics

For all statistical analyses, confirm that the following items are present in the figure legend, table legend, main text, or Methods section.

n/a	Confirmed	
☐	☒	The exact sample size (*n*) for each experimental group/condition, given as a discrete number and unit of measurement
☐	☒	A statement on whether measurements were taken from distinct samples or whether the same sample was measured repeatedly
☐	☒	The statistical test(s) used AND whether they are one- or two-sided<br>*Only common tests should be described solely by name; describe more complex techniques in the Methods section.*
☒	☐	A description of all covariates tested
☐	☒	A description of any assumptions or corrections, such as tests of normality and adjustment for multiple comparisons
☐	☒	A full description of the statistical parameters including central tendency (e.g. means) or other basic estimates (e.g. regression coefficient) AND variation (e.g. standard deviation) or associated estimates of uncertainty (e.g. confidence intervals)
☒	☐	For null hypothesis testing, the test statistic (e.g. *F*, *t*, *r*) with confidence intervals, effect sizes, degrees of freedom and *P* value noted<br>*Give P values as exact values whenever suitable.*
☒	☐	For Bayesian analysis, information on the choice of priors and Markov chain Monte Carlo settings
☒	☐	For hierarchical and complex designs, identification of the appropriate level for tests and full reporting of outcomes
☐	☒	Estimates of effect sizes (e.g. Cohen's *d*, Pearson's *r*), indicating how they were calculated

Our web collection on statistics for biologists contains articles on many of the points above.

Software and code

Policy information about availability of computer code

Data collection	Confocal images were collected by Zeiss software ZEN; Nissle staining were imaged by Keyence BZ9000; Electrophysiology data were collected by pClamp 9 Clampfit.
Data analysis	Image J, ZEN (blue edition), MiniAnalysis and Graphpad Prism 7.0 softwares were used.

For manuscripts utilizing custom algorithms or software that are central to the research but not yet described in published literature, software must be made available to editors/reviewers. We strongly encourage code deposition in a community repository (e.g. GitHub). See the Nature Research guidelines for submitting code & software for further information.

Data

Policy information about availability of data

All manuscripts must include a data availability statement. This statement should provide the following information, where applicable:

- Accession codes, unique identifiers, or web links for publicly available datasets
- A list of figures that have associated raw data
- A description of any restrictions on data availability

Original data sets are available upon reasonable request.

Field-specific reporting

Please select the one below that is the best fit for your research. If you are not sure, read the appropriate sections before making your selection.

☒ Life sciences ☐ Behavioural & social sciences ☐ Ecological, evolutionary & environmental sciences

Life sciences study design

All studies must disclose on these points even when the disclosure is negative.

Sample size	No statistical methods were applied to pre-determine sample size but our sample size are similar with previous published papers. (Guo et al., Cell stem Cell 2014; Dong et al., Nature Neuroscience 2014; Rivettl di Val Cervo et al., Nature biotechnology 2017).
Data exclusions	No data points were excluded from the analysis.
Replication	All of data in this study were collected from more than 3 independent experiments, and were reliably reproduced.
Randomization	Mice were selected randomly in this study.
Blinding	The experimenter was blind to the virus treatment when they performed immunostaining, behavior test and analysis.

Reporting for specific materials, systems and methods

We require information from authors about some types of materials, experimental systems and methods used in many studies. Here, indicate whether each material, system or method listed is relevant to your study. If you are not sure if a list item applies to your research, read the appropriate section before selecting a response.

Materials & experimental systems

n/a	Involved in the study	
☐	☒	Antibodies
☒	☐	Eukaryotic cell lines
☒	☐	Palaeontology
☐	☒	Animals and other organisms
☒	☐	Human research participants
☒	☐	Clinical data

Methods

n/a	Involved in the study	
☒	☐	ChIP-seq
☒	☐	Flow cytometry
☒	☐	MRI-based neuroimaging

Antibodies

Antibodies used	RFP (1:1000) Rat mAb Chromotek 5f8-100; NeuroD1 (1:1000) Mouse mAb Abcam AB60704; Dlx2 (1:1000) Rabbit Abcam AB30339 Discontinued; Dlx2 (1:200) Rabbit Millipore AB5726; Cre (1:1000) Mouse mAb Millipore MAB3120; GFAP (1:2000) Rabbit Millipore AB5804; GFAP (1:1000) Chicken Millipore AB5541; Glutamine synthetase (1:1000) Mouse Millipore MAB302; S100β (1:1000) Rabbit Abcam ab52642; NG2 (1:150) Mouse abcam ab50009; Olig2 (1:1000) Rabbit Millipore AB9610; Iba1 (1:1000) Rabbit Wako 019-19741; NeuN (1:2000) Guinea Pig Millipore ABN90; NeuN (1:2000) Rabbit Millipore ABN78; GAD67 (1:1000) Mouse Millipore MAB5406; GABA (1:1000) Rabbit Sigma A2052; DARPP32 (1:1000) Rabbit Millipore AB10518; Parvalbumin (1:5000) Mouse Sigma P3088; Somatostatin (1:300) Rat Millipore MAB354; NPY (1:2000) Rabbit Abcam AB30914; Calretinin (1:2000) Goat Millipore AB1550; vGAT (1:500) Guinea Pig SYSY 131004; mHtt (1:1000) Mouse mAb DSHB MW7; Ki67 (1:500)Rabbit abcam ab15580; S100β (1:1000) Mouse abcam ab66028.
Validation	All antibodies were selected according to the antibody validation which are available on the manufacturer's publicly accessible data sheets.

Animals and other organisms

Policy information about studies involving animals; ARRIVE guidelines recommended for reporting animal research

Laboratory animals	Both female and male adult mice (2-5 months) were used in this study. The R6/2 strain (B6CBA-Tg(HDexon1)62Gpb/3J) was maintained by ovarian transplant hemizygote females x B6CBAF1/J males, which were purchased from Jackson Laboratory. The littermates without mutation were used as the wild type. Some of the R6/2 mice (B6CBA-Tg(HDexon1)62Gpb/3J) were directly purchased from Jackson Laboratory when the mice age around 4-6 weeks. The GFAP::Cre transgenic mice (B6.Cg-Tg(Gfap::cre) 77.6Mvs/2J, Cre77.6) were also purchased from Jackson Laboratory.
Wild animals	No wild animals were used in this study.
Field-collected samples	The study did not involve samples collected from the field.
Ethics oversight	Our animal protocol were approved by the Pennsylvania State University IACUC and in accordance with guidelines of National Institutes of Health.

Note that full information on the approval of the study protocol must also be provided in the manuscript.
